# Species of *Philometra* (Nematoda, Philometridae) from fishes off the Mediterranean coast of Africa, with a description of *Philometra rara* n. sp. from *Hyporthodus haifensis* and a molecular analysis of *Philometra saltatrix* from *Pomatomus saltatrix*

**DOI:** 10.1051/parasite/2017008

**Published:** 2017-03-10

**Authors:** František Moravec, Amira Chaabane, Lassad Neifar, Delphine Gey, Jean-Lou Justine

**Affiliations:** 1 Institute of Parasitology, Biology Centre of the Czech Academy of Sciences Branišovská 31 370 05 České Budějovice Czech Republic; 2 Laboratoire de Biodiversité et Écosystèmes Aquatiques, Faculté des Sciences de Sfax (FSS), Université de Sfax BP 1171 3038 Sfax Tunisia; 3 Service de Systématique moléculaire, UMS 2700 CNRS, Muséum National d’Histoire Naturelle, Sorbonne Universités CP 26, 43 rue Cuvier 75231 Paris Cedex 05 France; 4 ISYEB, Institut Systématique, Évolution, Biodiversité, UMR7205 CNRS, EPHE, MNHN, UPMC, Muséum National d’Histoire Naturelle, Sorbonne Universités CP51, 55 rue Buffon 75231 Paris Cedex 05 France

**Keywords:** Parasitic nematode, Taxonomy, Dracunculoidea, Mediterranean Sea, Libya, Tunisia, COI, Barcoding

## Abstract

Two gonad-infecting species of *Philometra* Costa, 1845 (Nematoda, Philometridae) were recorded for the first time from marine perciform fishes off Tunisia and Libya: *Philometra rara* n. sp. from the rare, deep-water Haifa grouper *Hyporthodus haifensis* (Serranidae) off Libya and *Philometra saltatrix* Ramachandran, 1973 from the bluefish *Pomatomus saltatrix* (Pomatomidae) off Tunisia. Identification of both fish species was confirmed by molecular barcoding. Light and scanning electron microscope studies of *Ph. rara* n. sp. showed that it is characterized by the length of spicules (216–219 μm) and the gubernaculum (90–93 μm), the gubernaculum/spicules length ratio (1:2.32–2.43), and mainly by the shape and structure of the distal end of the gubernaculum (shovel-shaped with a wide median smooth field in dorsal view), appearing as having a dorsal protuberance in lateral view, and by the structure of the male caudal mound (dorsally interrupted); large subgravid females (70–137 mm long) are characterized by the presence of four oval submedian cephalic elevations, each of them bearing a pair of cephalic papillae of the outer circle. The finding of *Ph. saltatrix* off Tunisia confirms that this species is widespread throughout the Mediterranean region. A molecular analysis of our *Ph. saltatrix* specimens and other available philometrid cytochrome c oxidase 1 (COI) sequences showed that most species have robust clades. Sequences of *Ph. saltatrix* from Tunisia diverge from *Ph. saltatrix* from Brazil and the USA, suggesting that speciation is currently occurring between populations from both sides of the Atlantic Ocean.

## Abstract (fr)

Deux espèces de *Philometra* Costa, 1845 (Nematoda, Philometridae) parasites des gonades sont, pour la première fois, signalées de poissons perciformes marins au large de la Tunisie et de la Libye : *Philometra rara* n. sp. (Serranidae) du mérou de Haïfa *Hyporthodus haifensis*, une espèce rare, au large de la Libye et *Philometra saltatrix* Ramachandran, 1973, du tassergal *Pomatomus saltatrix* (Pomatomidae) au large de la Tunisie. L’identification des deux espèces de poissons a été confirmée par barcode moléculaire. Des études en microscopie photonique et microscopie électronique à balayage de *Ph. rara* n. sp. ont montré que l’espèce se caractérise par la longueur des spicules (216-219 μm) et du gubernaculum (90-93 μm), le rapport gubernaculum/spicules (1: 2.32-2.43) et principalement par la forme et la structure de l’extrémité distale du gubernaculum (en forme de pelle avec un large champ médian lisse en vue dorsale), apparaissant comme ayant une protubérance dorsale en vue latérale, et par la structure du monticule caudal mâle (interrompu dorsalement); les grandes femelles subgravides (70-137 mm de long) se caractérisent par la présence de quatre élévations céphaliques ovales sous-médianes, chacune portant une paire de papilles céphaliques sur le cercle extérieur. La découverte de *Ph. saltatrix* au large de la Tunisie confirme que cette espèce est répandue dans la région méditerranéenne. Une analyse moléculaire de nos spécimens de *Ph. saltatrix* et d’autres séquences disponibles de COI de Philometridae montre que la plupart des espèces présentent des clades robustes. Les séquences de *Ph. saltatrix* en provenance de Tunisie divergent de *Ph. saltatrix* du Brésil et des États-Unis, ce qui suggère qu’une spéciation se produit actuellement entre les populations des deux côtés de l’océan Atlantique.

## Introduction

To date, four nominal species of the nematode genus *Philometra* Costa, 1845 are known from marine fishes off the Mediterranean coast of North Africa, all parasitizing the gonads of groupers (Serranidae): *Ph. aenei* Moravec, Chaabane, Neifar, Gey and Justine, 2016 from *Epinephelus aeneus* (Geoffroy Saint-Hilaire) off Tunisia; *Ph. inexpectata* Moravec, Chaabane, Justine and Neifar, 2016 from *Mycteroperca rubra* (Bloch) off Tunisia; *Ph. jordanoi* (López-Neyra, 1951) from *Epinephelus marginatus* (Lowe) off Morocco and Tunisia; and *Ph. tunisiensis* Moravec, Chaabane, Neifar, Gey and Justine 2016 from *E*. *costae* (Steindachner) off Tunisia [11, 26, 27]. During recent helminthological investigations of some marine fishes in the Mediterranean Sea off the coast of Libya and Tunisia, an additional two species of *Philometra* were recorded, one known and one new to science. Results of their study are presented herein.

The Haifa grouper *Hyporthodus haifensis* (maximum body length 110 cm, maximum weight 25 kg) is a deep-water subtropical fish distributed in the Eastern Atlantic (Mediterranean to southern Angola) [6]. Other parasites (diplectanid monogeneans) were recently reported from *H. haifensis* from the same locality [1]. The bluefish *Pomatomus saltatrix* (maximum body length 130 cm, weight up to 14.4 kg) is an important commercial fish and game fish with a circumglobal distribution in tropical and subtropical waters [6].

Molecular analysis of *Philometra* species has concerned only a small number of species [4, 28, 33, 34]; sequences of cytochrome c oxidase 1 (COI), typically used for barcoding in other animals [30], were demonstrated to be effective in identifying philometrid species [4, 34]. In our study, we obtained sequences of COI from several *Ph. saltatrix* specimens and compared them with available sequences from other species.

## Materials and methods

### Fish and their identification

Fish were purchased at fish markets in Tunis and Sfax, Tunisia; these were previously caught by fishermen in the nearby coastal waters of the Mediterranean Sea.

**Figure 1.**
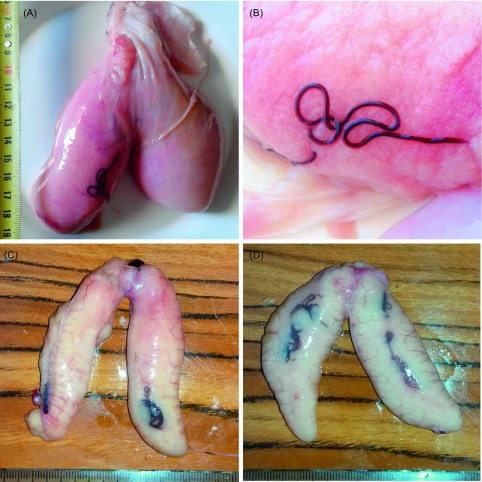
Ovaries of fish with visible *Philometra* females. (A, B) *Hyporthodus haifensis* with *Philometra rara* n. sp. (A) intact ovaries; (B) ovary with tunica removed. (C, D) *Pomatomus saltatrix* with *Philometra saltatrix*, two specimens. Scales: millimetres.

A single, whole specimen of Haifa grouper, *Hyporthodus haifensis* (Ben-Tuvia) (Serranidae), with mature gonads (Fig. 1) was caught from off Libya according to fishmongers of the Sfax fishmarket, Tunisia. Identification of the fish was performed according to usual keys and books [6, 12] and was confirmed by barcoding.

Specimens of bluefish, *Pomatomus saltatrix* (Linnaeus) (Pomatomidae), were obtained from the Tunis fishmarket (Fig. 1). Two fish specimens were identified from both morphology [6, 12] and barcoding (Table 1), and most others were identified solely on barcoding of separate organs (Table 1); see Results for details. The fish nomenclature adopted follows FishBase [6].

**Table 1.** *Pomatomus saltatrix*: fish gonad collected and fish identification.

Alleged fish species (according to fishmongers)	Material	Fish code	GenBank	Fish species (according to BOLD)	Percentage of similarity to *Po. saltatrix* (according to BOLD)	Philometrids collected	Date
“*Mugil cephalus*”*	Gonad only	Muce5	KY500055	*Pomatomus saltatrix*	99.83	None	08/09/2015
“*Mugil cephalus*”*	Gonad only	Muce6	KY500056	*Pomatomus saltatrix*	99.85	>20 Males, 1 female, 6 female fragments	08/09/2015
“*Mugil cephalus*”*	Gonad only	Muce7	KY500057	*Pomatomus saltatrix*	99.85	>100 Males, 1 female, 1 female fragment	08/09/2015
“*Mugil cephalus*”*	Gonad only	Muce8	KY500058	*Pomatomus saltatrix*	99.68	>20 Males, 6 females or fragments	08/09/2015
*Pomatomus saltatrix*	Whole fish	Posa5	KY500059	*Pomatomus saltatrix*	99.85	>20 Males, 9 whole females	09/09/2015
*Pomatomus saltatrix*	Whole fish	Posa6	KY500060	*Pomatomus saltatrix*	99.69	>50 Males, female fragments	09/09/2015
*Pomatomus saltatrix*	Whole fish	Posa7	No			>75 Males	27/09/2015
“*Micromesistius poutassou*”*	Gonad only	Mipou1	KY500061	*Pomatomus saltatrix*	99.69	>20 Males, several females	11/09/2015
“*Micromesistius poutassou*”*	Gonad only	Mipou2	KY500062	*Pomatomus saltatrix*	100	>20 Males, several females	11/09/2015
“*Micromesistius poutassou*”*	Gonad only	Mipou3	KY500063	*Pomatomus saltatrix*	100	>20 Males, several females	11/09/2015
“*Micromesistius poutassou*”*	Gonad only	Mipou4	KY500064	*Pomatomus saltatrix*	100	>50 Males, several females	11/09/2015
“*Micromesistius poutassou*”*	Gonad only	Mipou5	KY500065	*Pomatomus saltatrix*	99.84	>20 Males, several females	11/09/2015

Fish coded Posa5 and Posa6 were identified by both morphology and barcoding;

* : erroneous identifications by fishmongers.

### Barcoding of fish

Fish DNA was extracted from tissue (gonad) samples using the NucleoSpin 96 tissue kit (Macherey-Nagel, Düren, Germany) following the manufacturer’s instructions. Sequences were obtained by amplification and sequencing of a region of the cytochrome c oxidase subunit I (COI) mitochondrial gene using the primers FishF1 (5’-TCAACYAATCAYAAAATYGGCAC-3’) and FishR1 (5’-TGATTYTTYGGYCACCCRGAAGT-3’) [35]. Standard Polymerase chain reactions (PCRs) were carried out in 20 μL total volume, containing about 30 ng of DNA, 1 × 10x PCR buffer, 2 mM MgCl_2_, 200 μM mix dNTPs, 150 nM of each primer and 1 unit of Taq polymerase (Qiagen, Hilden, Germany). After an initial denaturation of 3 min at 95 °C, amplification was performed through 39 cycles of 15 s at 95 °C, 20 s at 48 °C, and 40 s at 72 °C, with a terminal elongation for 5 min at 72 °C. PCR products were purified and sequenced in both directions on 3730xl DNA Analyzer 96-capillary sequencer (Applied Biosystems, Waltham, MA, USA). Sequences were edited using CodonCode Aligner software (CodonCode Corporation, Dedham, MA, USA), compared with the GenBank database content using BLAST, and deposited in GenBank under accession numbers KY500054–KY500065. Species identification was confirmed using the BOLD identification engine [30]. Since BOLD does not include all sequences available in GenBank but includes others, comments are added for similarities with other sequences.

### Collection of philometrids

Philometrid specimens were collected from fresh (Haifa grouper) or frozen-thawed (Bluefish) gonads under the dissecting microscope. They were fixed in hot 70% ethanol and processed for examination or molecular techniques.

### Barcoding of philometrids

The same method was used for female fragments (ca. 1–2 mm^3^) and individual whole males. Genomic DNA was extracted using the QIAamp DNA Micro Kit (Qiagen). A ≈ 400 bp fragment of the mitochondrial cytochrome c oxidase I (COI) gene was amplified with the nematode-specific PCR primers NemCOI5P (CATTTRTTTTGRTTTTTTGG) and NemCOI3P (ACYACATRATAAGTATCRTG) [4]. PCRs were performed in a final volume of 20 μL, containing 1.5 μL isolated DNA, 1 × CoralLoad PCR buffer, 3 mm MgCl_2_, 66 μM of each dNTP, 0.15 μM of each primer and 0.5 units of Taq DNA polymerase (Qiagen). The amplification protocol was: 94 °C for 4 min followed by two cycles at 94 °C for 30 s, 45 °C for 30 s, and 72 °C for 30 s, then 40 cycles at 94 °C for 30 s, 55 °C for 30 s, and 72 °C for 30 s, with a final extension at 72 °C for 5 min. PCR products were purified and sequenced in both directions on 3730xl DNA Analyzer 96-capillary sequencer (Applied Biosystems). Sequences were edited using CodonCode Aligner software (CodonCode Corporation, Dedham, MA, USA), compared to the GenBank database content using BLAST, and deposited in GenBank under accession numbers KY500066–KY500070.

### Trees and distances

A tree was constructed from most available COI sequences of philometrids, including sequences already available in GenBank and our new sequences. The analysis involved 36 nucleotide sequences and there were a total of 234 positions in the final dataset. The tree, computed in MEGA7 [9] with 1000 bootstrap replications [5], was inferred using the Neighbour-Joining method [32] and Kimura-2 parameter distance [8]. *Clavinema* sp. was set as the outgroup. Genetic distances (Kimura-2 parameter distance [8]) were estimated with MEGA7 [9]. All codon positions were used.

### Description of philometrids

Philometrid specimens were cleared with glycerine for light microscope (LM) examination. Drawings were made with the aid of a Zeiss drawing attachment. Specimens used for scanning electron microscopy were postfixed in 1% osmium tetroxide (in phosphate buffer), dehydrated through a graded acetone series, critical-point-dried and sputter-coated with gold; they were examined using a JEOL JSM-7401F scanning electron microscope at an accelerating voltage of 4 kV (Gentle Beam (GB) low mode). All measurements are in micrometers unless indicated otherwise.

## Results

### Molecular study of host fish

#### Haifa grouper, *Hyporthodus haifensis*

The sequence of our specimen (KY500054), submitted to a BLAST in GenBank, showed 100% similarity with three sequences of the same species collected off Libya and Tunisia [1], and, in BOLD [30], showed 100% similarity with sequences of the same species from off Sicilia, Italy [10]. This confirms the identification of the species.

#### Bluefish, *Pomatomus saltatrix*

Whole specimens of Bluefish with mature gonads were purchased and were barcoded. When asked for more mature gonads showing visible philometrid females, fishmongers of the Tunis fishmarket were keen to provide to one of us (AC) selected infected gonads, *allegedly from several fish species*. The day after, the gonads were examined at the fishmarket, chosen for the presence of female philometrids and purchased, but they were sold separated from the fish; they were processed for parasitological examination and a tissue sample was taken for each gonad. After barcoding (Table 1), it was found that all fish gonads in fact belonged to a single species, *Pomatomus saltatrix*.

### Morphology of philometrids

#### *Philometra rara* n. sp. Figures 2–4

urn:lsid:zoobank.org:act:286AFEC6-0888-4643-BDE4-ABB0952F17A4

**Figure 2.**
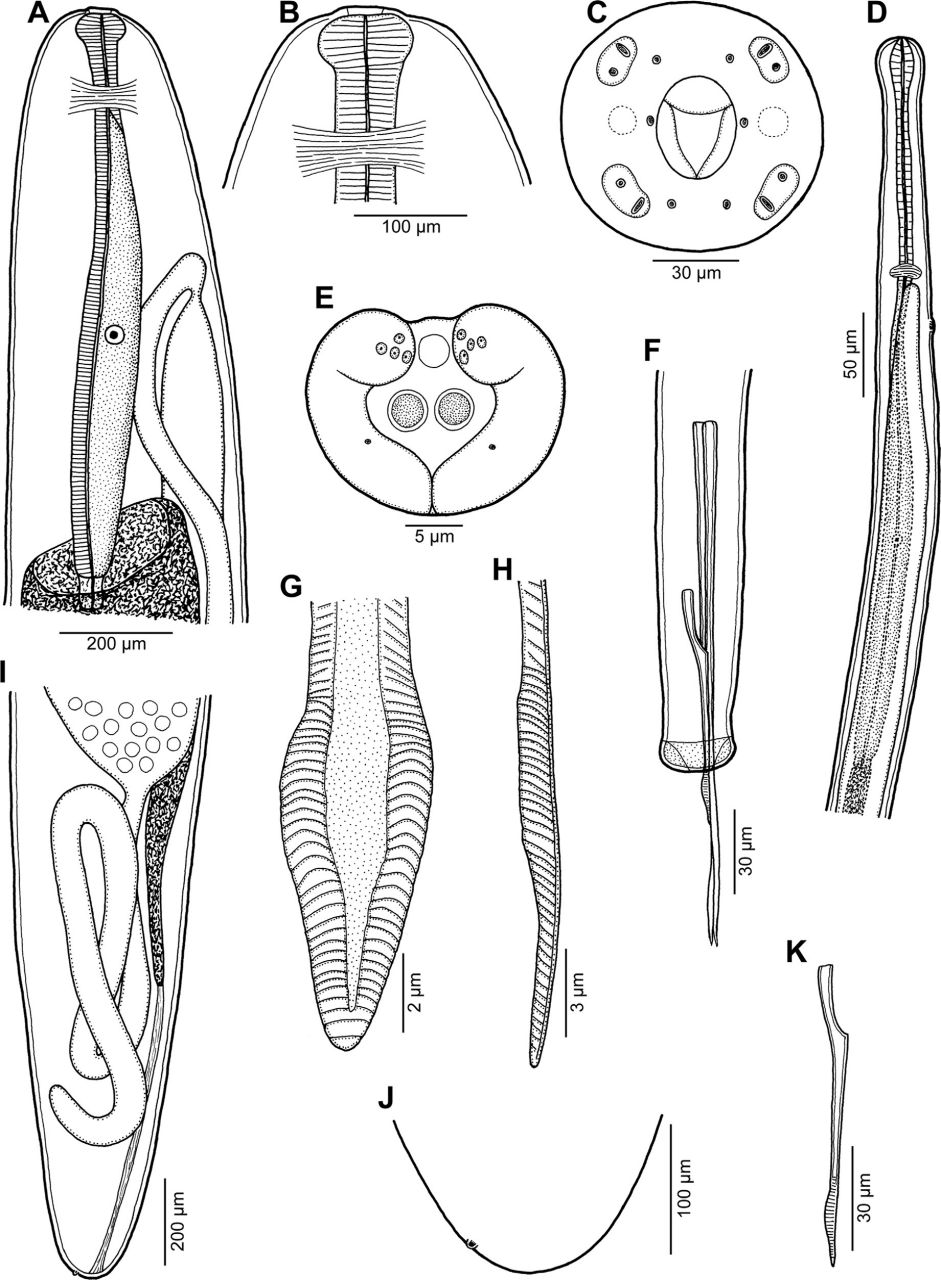
*Philometra rara* n. sp. (A) anterior end of subgravid female, lateral view; (B, C) cephalic end of subgravid female, lateral and apical views; (D) anterior end of male, lateral view; (E) caudal end of male, apical view; (F) posterior end of male, lateral view; (G, H) posterior end of gubernaculum, dorsal and lateral views; (I) posterior end of subgravid female, lateral view; (J) caudal end of subgravid female, lateral view; (K) gubernaculum, lateral view.

Type host: Haifa grouper *Hyporthodus haifensis* (Ben-Tuvia) (Serranidae, Perciformes). Molecular identification of host confirmed morphological identification (see above).

Site of infection: Gonad (Fig. 1).

Type locality: From fishmarket in Sfax (Tunisia), allegedly from off Libya (collected 5 August 2016).

Prevalence and intensity: 1 fish infected/1 fish with mature gonads examined; 4 nematode specimens per fish.

Deposition of type specimens: Holotype (male) and allotype (female), Muséum National d’Histoire Naturelle, Paris, MNHN HEL594–HEL595; 1 female paratype (specimen without body ends) in the Helminthological Collection of the Institute of Parasitology, Biology Centre of the Czech Academy of Sciences, České Budějovice (Cat. No. N–1128).

Etymology: The specific name *rara* is a Latin adjective (*rarus* = rare, seldom) and relates to a rather rare occurrence of its fish host.

**Figure 3.**
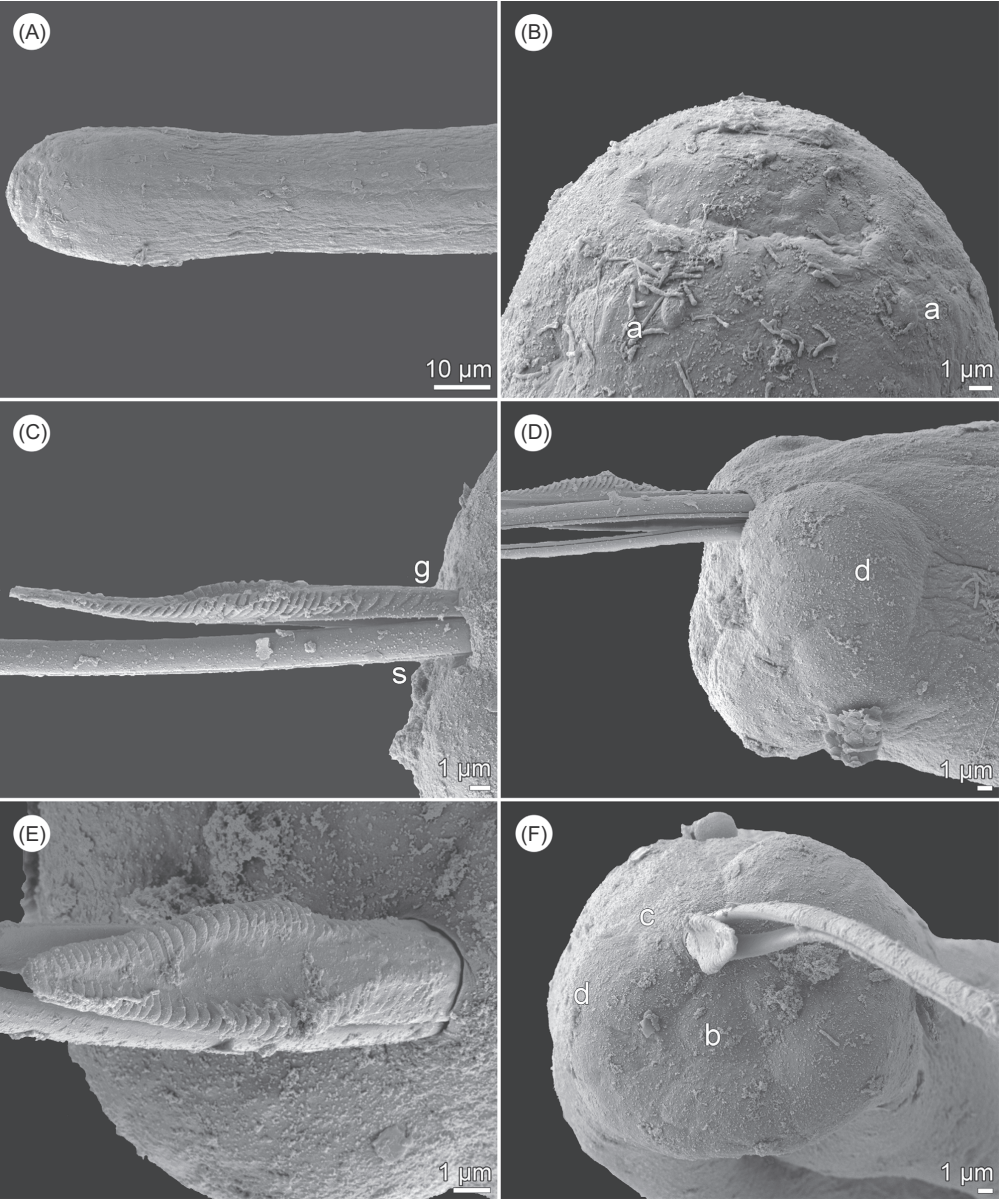
*Philometra rara* n. sp., scanning electron micrographs. (A) Anterior end of male; (B) cephalic end of male, dorsoventral view; (C) gubernaculum, lateral view; (D) caudal end of male, lateral view; (E) gubernaculum, dorsal view; (F) caudal end of male, apical view. Abbreviations: (a) submedian pair of outer cephalic papillae; (b) dorsal caudal papilla; (c) group of four adanal papillae; (d) caudal mound; (g) gubernaculum; (s) spicule.

**Figure 4.**
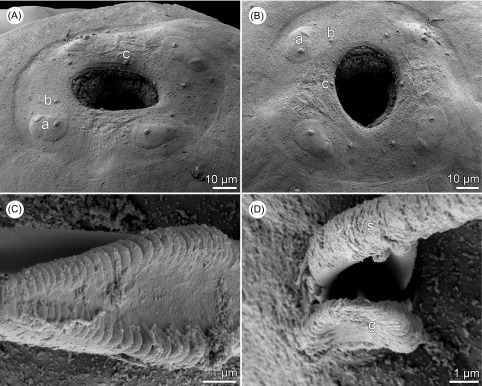
*Philometra rara* n. sp., scanning electron micrographs. (A, B) Cephalic end of subgravid female, dorsoventral and apical views, respectively; (C) posterior end of gubernaculum, dorsal view; (D) apical view of posterior end of gubernaculum with two distinct ventral longitudinal grooves.

*Description of male* (2 specimens; holotype; measurements of paratype in parentheses): Body filiform, whitish, 2.19 (2.27) mm long, maximum width at middle of body 51 (60); anterior part of body somewhat constricted just posterior to cephalic end (Figs. 2D, 3A); body width at this constriction 21 (27). Maximum width/body length ratio 1:43 (1:38). Cuticle smooth. Cephalic end rounded, 27 (33) wide. Morphology of cephalic end probably identical to that in other congeners, i.e., small oral aperture surrounded by 14 cephalic papillae arranged in two circles and by pair of small lateral amphids; outer circle of cephalic papillae formed by four submedian pairs (Fig. 3B); inner circle formed by four submedian and two lateral papillae, amphids and oral aperture not visible on available SEM micrograph (Fig. 3B). Oesophagus 456 (570) long, comprising 21% (25%) of body length, with slight inflation at anterior end measuring 27 × 15 (33 × 21); posterior part of muscular oesophagus overlapped by well-developed oesophageal gland with large cell nucleus; maximum width of gland 21 (24). Nerve ring and oesophageal nucleus 144 (135) and 306 (306), respectively, from anterior extremity. Excretory pore 165 (159) from anterior end. Testis extending anteriorly to level of nerve ring (Fig. 2D), overlapping posterior portion of oesophagus. Posterior end of body blunt, 27 (30) wide, with broad caudal mound consisting of two reniform lateral parts broadly separated from each other ventrally and adhering to each other dorsally (Figs. 2E, 3D, 3F). Four adanal pairs of small, very flat, hardly visible caudal papillae present on anterior parts of caudal mound; additional pair of large subdorsal papillae situated dorsally to cloacal aperture (Figs. 2E, 3D, 3F). Pair of small phasmids present slightly posterior to middle of each part of caudal mound in apical view (Fig. 2E). Spicules slender, needle-like, equally long, with somewhat expanded proximal and sharply pointed distal tips (Figs. 2F, 3C, 3D, 3F); length of spicules 219 (216), representing 10% (10%) of body length. Gubernaculum 90 (93) long, with anterior portion somewhat dorsally bent; length of anterior bent part 30 (30), representing 33% (32%) of entire gubernaculum length (Figs. 2F–2H, 2F, 3C–3F); distal end of gubernaculum with dorsal protuberance and numerous dorsolateral transverse lamella-like structures; dorsal protuberance on gubernaculum appears as single in lateral view (Figs. 2F, 2H, 2K, 3C, 3D) but, in fact, it consists of two dorsolateral parts separated from each other by wide, smooth longitudinal field when observed dorsally (Figs. 2G, 2H, 3C–3F, 4C, 4D); distal end of gubernaculum shovel-shaped in dorsal view (Figs. 2G, 3E, 4C) and with two ventral longitudinal grooves (Fig. 4D). Length ratio of gubernaculum and spicules 1:2.43 (1:2.32). Spicules and gubernaculum well sclerotized, yellowish, anterior part of gubernaculum colourless.

*Subgravid female* (two ovigerous specimens; allotype; measurements of paratype in parentheses): Body of fixed specimen brown, with distinct dark-brown intestine visible through cuticle, ends rounded. Posterior part of body somewhat narrower than anterior part; maximum width in region just posterior to oesophagus. Cuticle smooth. Body length 70 (137) mm, maximum width 694 (884), maximum width/body length ratio 1:101 (1:155). Width of cephalic end 218 (245). Cephalic papillae small, indistinct when viewed laterally (Figs. 2A, 2B). Oral aperture oval, surrounded by small cephalic papillae arranged in two circles and slightly outlined amphids; inner circle of papillae consists of four submedian and two lateral single papillae, outer circle formed by four submedian pairs of papillae, each pair composed of one short and one elongate papilla; each submedian pair of outer papillae located on distinct oval cuticle elevation (Figs. 2C, 4A, 4B). Oesophagus including well-developed anterior bulbous inflation 1047 (870) long, comprising 1.5% (0.6%) of body length; anterior inflation 57 (66) long and 99 (135) wide; maximum width of posterior part of oesophagus including gland 90 (122). Oesophageal gland well developed, opening into oesophagus just posterior to nerve ring, with large cell nucleus at middle (Fig. 3A). Nerve ring and oesophageal nucleus 204 (218) and 653 (530), respectively, from anterior extremity. Ventriculus small, 41 (27) long, 82 (51) wide. Intestine brown, straight, ending blindly; anterior end of intestine wide; posterior end of intestine atrophied, forming ligament 462 (585) long attached ventrally to body wall close to posterior extremity (Fig. 2I). Vulva and anus absent. Ovaries reflexed near body ends (Figs. 2A, 2I). Uterus occupying most space of body, filled with numerous eggs (Fig. 2I). Posterior end of female rounded, 204 (190) wide, with two small lateral papilla-like caudal projections (Figs. 2I, 2J).

##### Remarks

Gonad-infecting species of *Philometra* are known to exhibit a high degree of host specificity [20, 21, 23, 26, 27]. Therefore, *Ph. rara* n. sp. is compared with the 16 other gonad-infecting nominal species of this genus described from fishes of the perciform family Serranidae: *Ph. aenei*; *Ph. cephalopholidis* Moravec and Justine, 2015; *Ph. charlestonensis* Moravec, de Buron, Baker and González-Solís, 2008; *Ph. cyanopodi* Moravec and Justine, 2008; *Ph. fasciati* Moravec and Justine, 2008; *Ph. hyporthodi* Moravec and Bakenhaster, 2013; *Ph. incognita* Moravec and Bakenhaster, 2016; *Ph. indica* Moravec and Manoharan, 2014; *Ph. inexpectata*; *Ph. jordanoi*; *Ph. margolisi* Moravec, Vidal-Martínez and Aguirre-Macedo, 1995; *Ph. mexicana* Moravec and Salgado-Maldonado, 2007; *Ph. piscaria* Moravec and Justine, 2014; *Ph. serranellicabrillae* Janiszewska, 1949; *Ph. tropica* Moravec and Manoharan, 2014; and *Ph. tunisiensis* (see Moravec et al. [26]). Five of them, *Ph. aenei*, *Ph. inexpectata*, *Ph. jordanoi*, *Ph. serranellicabrillae* and *Ph. tunisiensis*, occur in the Mediterranean region [7, 14, 26, 27].

Moravec et al. [26] have recently published the key to all these gonad-infecting species of *Philometra* parasitizing serranid fishes, based on morphological and biometrical features. According to the key, *Ph. rara* n. sp. is closest to *Ph. jordanoi*, a specific parasite of dusky grouper *Epinephelus marginatus* (Lowe) in the Mediterranean region [11, 13, 14, 18, 24, 26]; the male of *Ph. jordanoi* was redescribed in detail by Moravec et al. [26]. Both these species resemble each other by the length of spicules (216–219 μm in *Ph. rara*, 213–252 μm in *Ph. jordanoi*), the number and distribution of male caudal papillae, the approximate number (about 30) of transverse lamellar structures on the gubernaculum distal tip and by the ventral surface of the posterior end of the gubernaculum with two ventral longitudinal grooves. However, *Ph. rara* differs distinctly from *Ph. jordanoi* in the shape and structure of the distal end of the gubernaculum (shovel-shaped with a wide median smooth field *vs*. tongue-shaped with a narrow smooth field in dorsal view; appearing as having a distinct dorsal protuberance *vs*. without such a protuberance in lateral view). Whereas the caudal mound of *Ph. rara* is dorsally interrupted, that of *Ph. jordanoi* is V-shaped, dorsally uninterrupted. The former species also differs somewhat from the latter in the male body length (2.19–2.27 mm *vs*. 2.45–2.91 mm), the presence (*vs*. absence) of a body constriction just behind the male cephalic extremity, the length of the gubernaculum (90–93 μm *vs*. 81–84 μm), the gubernaculum/spicules length ratio (1:2.32–2.43 *vs*. 1:2.61–3.11) and the relative length of spicules to the length of the body (10% *vs*. 8%).

The outer cephalic papillae of large females of *Ph. rara* are located on four distinct submedian cuticular elevations as well as in *Ph. jordanoi* (as described by Moravec et al., 2003 [24]) or, for example, in *Ph. fasciati* (reported by Moravec and Justine, 2014 [19]). However, each pair of these papillae is formed by one circular and one elongate-oval papilla in apical view in *Ph. rara*, in contrast to that of *Ph. jordanoi* or *Ph. fasciati*, in which the pair is formed by two circular papillae in apical view. The female caudal end of *Ph. rara* bears a pair of small lateral papilla-like projections, like e.g. that of *Ph. fasciati*, but no female caudal projections were reported for *Ph. jordanoi*.

To date, only one gonad-infecting species of *Philometra* is known to parasitize hosts of the genus *Hyporthodus* Gill: *Ph. hyporthodi*, a parasite of *H*. *flavolimbatus* (Poey) in the northern Gulf of Mexico [15]. However, this species can be easily distinguished from *Ph. rara* n. sp. by the distinctly longer males (3.62–4.07 mm *vs*. 2.19–2.27 mm), shorter spicules (135–138 μm *vs*. 216–219 μm) comprising 4% (*vs*. 10%) of the body length, the caudal mound consisting of two lateral parts widely separated dorsally from each other and by the different shape and structure of the distal end of the gubernaculum. The female caudal projections are absent (*vs*. present).

#### *Philometra saltatrix* Ramachandran, 1973

Host: Bluefish *Pomatomus saltatrix* (Linnaeus) (Pomatomidae, Perciformes). Molecular identification of hosts: see Table 1 and text.

Site of infection: Gonad (Fig. 1).

Locality: Off Mediterranean coast of Tunisia (fish market in Tunis and Sfax) (collected September 2015).

Prevalence and intensity: 96% (25 fish infected/26 fish with mature gonads or mature gonads alone examined); 24–112 (mean 54) nematode specimens per fish.

Deposition of voucher specimens: Muséum National d’Histoire Naturelle, Paris (MNHN HEL596); Helminthological Collection of the Institute of Parasitology, Biology Centre of the Czech Academy of Sciences, České Budějovice (Cat. No. N–809).

##### Remarks about morphology

The present material consisted mostly of nematode males, whereas nongravid and subgravid (ovigerous) females (maximum body length of about 80 mm) were much less numerous; no gravid (larvigerous) females were present. The morphology of available specimens was in full agreement with the redescriptions of *Ph. saltatrix* provided by Moravec et al., 2008 [25] and Moravec and de Buron [16].

*Philometra saltatrix* is a parasite of the bluefish *Po. saltatrix*, from which it was reported in North America (Northwest Atlantic, USA: off New York, North Carolina and South Carolina) [2, 3, 16, 22, 29] and in the Mediterranean Sea (Iskenderun Bay off Turkey and Tuscan Sea off Italy) [18, 25]. The present record of *Ph. saltatrix* from Tunisian waters shows that this parasite is widespread in the Mediterranean Sea. Conspecific with *Ph. saltatrix* is probably also *Philometra* sp. reported by Rego et al. [31] from *Ph. saltatrix* off the Brazilian coast (see [16]). *Philometra saltatrix* appears to be a specific parasite of *Pomatomus saltatrix* throughout the distribution area of this fish; however, our molecular results suggest that differences exist between specimens from both sides of the Atlantic Ocean.

### Molecular study of philometrids

Sequences of *Ph*. *saltatrix* were obtained for five female fragments out of five (100% success). No DNA was obtained from three males, probably because males are much smaller; no modification of the protocol was attempted for males.

**Figure 5.**
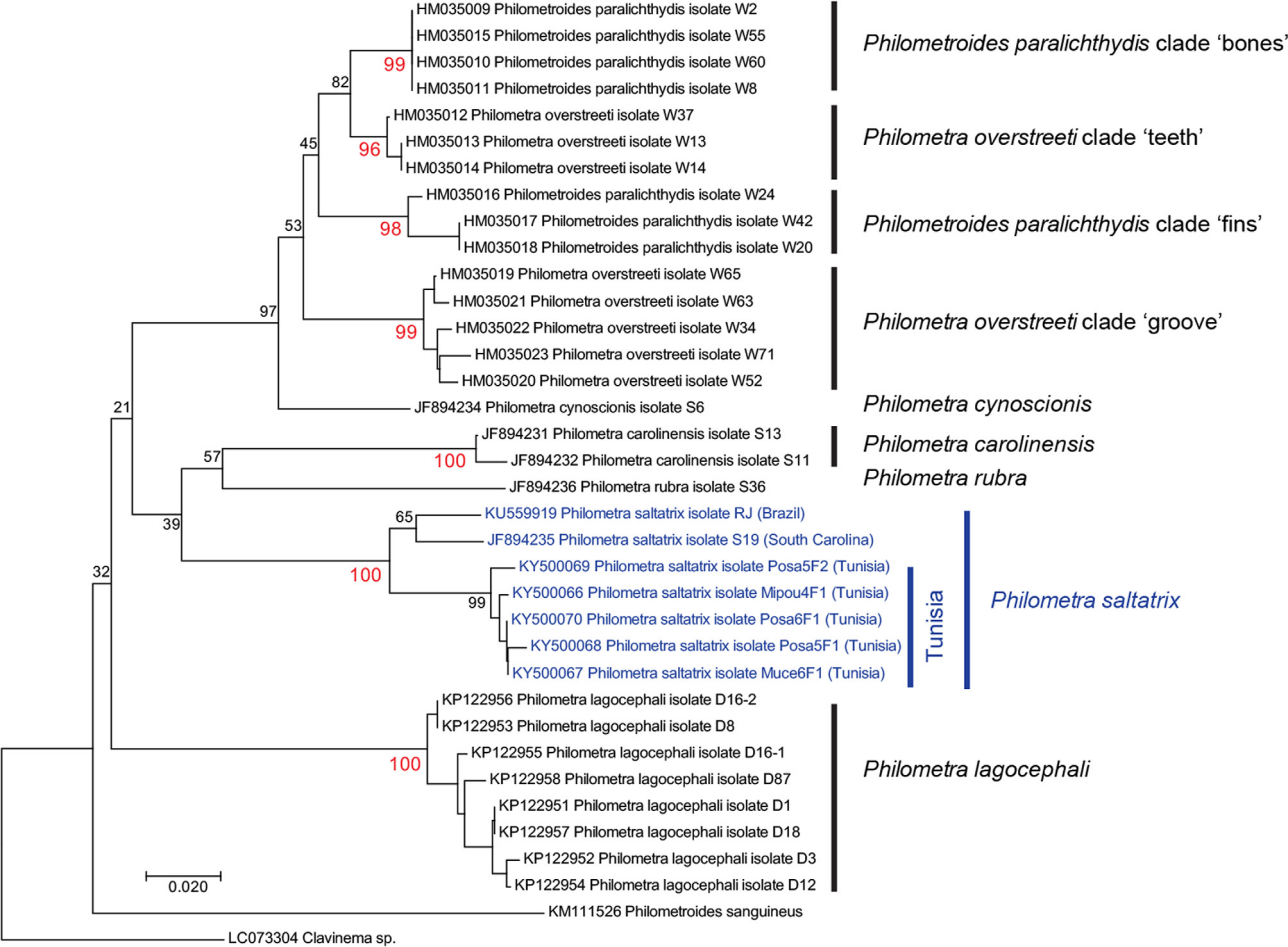
Tree of philometrids based on COI sequences. The evolutionary history was inferred using the Neighbour-Joining method. Bootstrap test results are shown next to the branches; red: bootstrap for clades corresponding to nominal species or clades within species (for *Philometroides paralichthydis* and *Philometra overstreeti*). For *Philometra saltatrix*, all specimens are within a well-supported clade but specimens from Tunisia are separated from specimens from the Western Atlantic (Brazil and South Carolina).

The tree produced with the Neighbour-Joining (NJ) method (Fig. 5) included 36 sequences, including our five new sequences of *Ph. saltatrix* from Tunisia. All species of marine *Philometra* and *Philometroides* formed a clade (with, however, low support value, 32%) from which the freshwater species *Philometroides sanguineus* and *Clavinema* sp. were clearly separated (distances of these two to all others: 15.7–24.2%). Relationships between marine philometrid clades showed low support; however, this is of minor relevance since the purpose of our study was not to produce phylogenies but to identify robust clades and compare them with hypotheses regarding the validity of species. All clades identified in previous studies were robust in the analysis, with bootstrap values ranging from 96 to 100%. These included the four clades from the same fish species, the Southern flounder *Paralichthys lethostigma* (*Philometroides paralichthydis* clade “bones” and clade “fins” and *Ph. overstreeti* clade “teeth” and clade “groove”), *Ph. carolinensis* and *Ph. lagocephali*.

All seven sequences of *Ph. saltatrix* formed a robust clade (bootstrap 100%); within this clade, the five sequences from worms collected in fishes from Tunisia formed a robust clade (bootstrap 99%) but the two sequences of worms collected in fishes from Brazil and South Carolina were united in a low support clade, separated from the clade of the Tunisian sequences. The distance of the specimen from Brazil to the Tunisian specimens was 4.9–5.9%, of the specimen from South Carolina to Tunisia 6.8–6.8%, and the specimens from Brazil and South Carolina had a 3.5% distance. Distances between *Ph. saltatrix* (all sequences) and other clades ranged from 14.6 to 20.1%.

## Discussion

### Molecular study of hosts

Table 1 shows that correct and accurate identification of fish is paramount in parasitology; had we blindly believed the fishmongers, we would have reported several additional host species for *Ph. saltatrix*; barcoding proved that all fish gonads in fact belonged to a single species, *Po. saltatrix*.

### Molecular study of philometrids

Our tree (Fig. 4) confirms philometrid clades identified in previous molecular studies, which were performed when a smaller number of species were available. Particularly, the four clades identified by Palesse et al. [28], i.e. *Philometroides paralichthydis* clade “bones” and clade “fins” and *Ph. overstreeti* clade “teeth” and clade “groove”, all from the same fish, the Southern flounder off South Carolina, are recognized. In each of these clades, the distances between members of each clade ranged from 0 to 2.2%. These distances are similar to what is found in *Ph. carolinensis* (intra-distance 0.4%) and *Ph. lagocephali* (intra-distance 0.2–2%). All these clades might probably be considered as four separate species, even though the four clades from the Southern flounder have not received separate binomials [28].

Our results for *Ph. saltatrix* are puzzling. All five specimens from the Tunisian clade show intra-distances from 0.4 to 1.3%, which correspond to the intra-distances found in other species (Table 2); however, distances within all specimens of the species, including specimens from the Western Atlantic and the Mediterranean Sea, show values from 0.4 to 6.8% (Table 2). This suggests that (a) all specimens from Tunisia belong to a single species; and (b) specimens from the Western Atlantic and the Mediterranean might belong to different species. Specimens from both sides of the Atlantic (the Americas *vs*. the Mediterranean Sea) were from fish of the same species, *Pomatomus saltatrix*; our results suggest that the geographical populations of the parasite *Ph. saltatrix* are currently undergoing speciation. However, all specimens of *Ph. saltatrix* from all localities were grouped in a single clade with high support (100%). Hence, the case of *Ph. saltatrix* needs further investigation involving more specimens from more localities throughout the range of bluefish, *Po*. *saltatrix*.

**Table 2.** Intra-species distances (Kimura-2 parameter) calculated from COI sequences of philometrids.

Clade	Intraclade distance (%)
*Philometroides paralichthydis* clade “bones”	0
*Philometra overstreeti* clade “teeth”	0–0.4
*Philometroides paralichthydis* clade “fins”	0–1.7
*Philometra overstreeti* clade “groove”	0.4–2.2
*Philometra carolinensis*	0.4
*Philometra lagocephali*	0–2.2
*Philometra saltatrix* (all localities)	0.4–6.8
*Philometra saltatrix* (Tunisia)	0.4–1.3

For practical purposes, it remains that sequences of all philometrid species currently available show robust clades; this suggests that the use of barcoding may be effective in identifying philometrid species in the absence of morphological studies. This is of particular importance when only female fragments are available. As already emphasized in 2011 by Palesse et al. [28], the molecular database is still extremely limited (currently nine species or clades) in comparison to the number of philometrid species currently accepted (more than 150 [17]).

## Conflict of interest

The Editor-in-Chief of Parasite is one of the authors of this manuscript. COPE (Committee on Publication Ethics, http://publicationethics.org), to which Parasite adheres, advises special treatment in these cases. In this case, the peer-review process was handled by an Invited Editor, Jerôme Depaquit.
